# Therapeutic Notes

**Published:** 1896-04-18

**Authors:** 


### THERAPEUTIC NOTES.

### HORSES' SERUM EMPLOYED AS A GENERAL TONIC IN CHILDHOOD.

Dr. José Mosequer Lacruz,[1] of Barcelona, has made a somewhat novel experiment in sero-therapeutics. Having prepared some serum from a healthy horse, he injected three to five cubic centimetres of it into a number of infants. The treatment was continued for three or four months. The number of the red corpuscles is said to have materially increased, the body weight was augmented, and the children regained their natural energies. The treatment appears to have been followed by no ill-effects either in the way of elevation of temperature or increased frequency of the pulse, erythema, or albuminuria, although a slight epithelial deposit was observed in the urine. The effect of the injections appears to be prompt, especially in cases of anæmia and general atrophic conditions. The author of the experiments is reported to be proceeding with his researches. The method of injecting food substances into the intercellular tissues is by no means a new one. In cases in which food could not be retained by any other method, we have seen good results follow the injection of Benger's peptone jelly; what special advantage is to be derived from the employment of horses' serum we are unable to see. It is difficult to obtain, especially in pure conditions; it cannot be sterilised by boiling as can peptone; neither will it keep for indefinite periods. Possibly the object for choosing this out-of-the-way material as a food or as a medicant may be explained in Dr. Lacruz's next paper.

[1] Archivos de Ginecopatia Ostetricia y Pediatria, Dec, 25, 1895.

### LOCAL ACTION OF SALOL.

For internal use the value of salol has long been recognised, owing partly to clinical results, and partly to the knowledge that within the intestine it breaks up into salicylic acid and phenol, both valuable antiseptics. In acid media, such as gastric juice, for instance, it is insoluble and inactive, and for this reason it is extensively used for the coating of pills, which are intended for intestinal use only, and which might do damage by dissolving in the stomach. In a paper by Colombini (Rif. Med., Sep., 1895), its local action on ulcers and inflamed surface is pointed out —salol undergoes decomposition not only in alkaline media, but in the presence of living tissues—the liberated antiseptics, in their nascent condition, appear to exercise no irritating properties, but under their influence unhealthy surfaces quickly heal. The acids are supposed to be slowly evolved and taken up by the tissue almost as quickly as they are formed, and hence no concentrated effect is produced. The constant, but slow, production of new quantities of an antiseptic is just what is wanted in the local treatment of certain skin diseases. Colombini recommends that the salol be dissolved in liquid vaseline, and applied directly to the damaged skin surfaces.

